# Business culture impairs facial trustworthiness judgments

**DOI:** 10.3389/fpsyg.2024.1356305

**Published:** 2024-05-01

**Authors:** Hongchuan Zhang, Yitong Liu, Weiran Li, Mengjie Nie, Ziqiang Xin

**Affiliations:** ^1^School of Sociology and Psychology, Central University of Finance and Economics, Beijing, China; ^2^School of Education, University of Glasgow, Glasgow, United Kingdom; ^3^Department of Psychology, Renmin University of China, Beijing, China

**Keywords:** business culture, trust, face recognition, trustworthiness judgment, *homo economics belief*

## Abstract

Previous research has found that business culture has a detrimental impact on interpersonal trust. To understand whether this impact extends to rapid, automatic, bottom–up judgments of facial trustworthiness, we conducted 4 experiments involving 244 participants from economic and non-economic backgrounds. We presented participants with both trustworthy and untrustworthy faces and asked them to make judgments on trustworthiness. The results show that individuals who are engaged in studying economics, work in an economics-related occupation, or are exposed to an imagined business culture evaluate trustworthy faces to be less trustworthy. The findings shed light on why and how business culture affects the formation of interpersonal trust.

## 1 Introduction

Trust plays a critical role in maintaining relationships between people, but there is increasing evidence showing that the level of interpersonal trust in the world has been declining dramatically with the rapid economic growth experienced by the country over the last several decades. For example, in the World Value Survey data, it was found that interpersonal trust decreased worldly between 1990 and 2001; and according to the Asian Barometer Survey, interpersonal trust in mainland China dropped by 18.5% from 1990 to 2002 (Ma, 2008). Recent analyses revealed that China has witnessed a sharp drop in interpersonal trust among the general population and college students (Xin and Zhou, 2012; Xin and Xin, 2017; Zhang and Xin, 2019; Yang and Xin, 2020). Researchers have linked this phenomenon to recent worldwide financial crises and have called for governments around the world to pay attention because trust is fundamental to financial transactions and industrial cooperation (Sapienza and Zingales, 2012).

Why is trust declining? One possible reason is that business culture has prevailed along with the trend of rapid economic growth. Business culture focuses on materialistic values and the pursuit of profit and exhibits a tolerance for unethical behaviors, which may lead to a crisis of trust (Smith, 2012). However, we cannot draw this causal conclusion by simply linking the declining level of trust with economic growth. One possible way is to compare those who have been more exposed to business culture, like economists, with those who have less exposure. Previous research has shown that economists behave differently from others in terms of pro-social behavior. For example, students studying economics behave more selfishly than students from other majors in third-party punishment games, being skeptical of fairness and preferring that everyone disobey fairness norms (Gerlach, 2017). Similarly, Ifcher and Zarghamee (2018) suggest that economics students exhibit more selfishness than students from other majors, as well as those non-economics majors who are even briefly exposed to common neoclassical economic assumptions significantly shift their behavior toward egoism. Research also indicated that business and economics majors are less averse to lies than students in other majors (López-Pérez and Spiegelman, 2019). In parallel, inspired by the economic theory of identity (Kielhofner, 2008; Akerlof and Kranton, 2010), Cohn et al. (2014) showed that when primed with their occupational identity, a significantly higher proportion of bank employees became dishonest in comparison to non-bank employees. Xin et al. (2013) compared the trust level of college students in their first year vs. their third year and found that students majoring in economics showed a significant decline in trust, a trend that was not found in students majoring in the humanities and social sciences. Further, Xin and Liu (2013) found that participants who were exposed to business culture while transcribing an introduction about economics exhibited significantly lower trust scores in comparison to those transcribing a paragraph of expository writing. Similarly, other studies suggested that individuals who were exposed to market relations or reminded of their shopping experiences exhibited proportional thinking, competitiveness, and self-interest (Bauer et al., 2012; Zaleskiewicz et al., 2020; Kuzminska et al., 2023). Therefore, when people are engaged in activities related to business culture, they may possess a “business” occupational identity. Occupational identity is the sense of who a person is and wishes to become in terms of his or her career (Kielhofner, 2008). Such identities are associated with specific social norms and lead to a shift in individuals' behaviors toward those norms (Shih et al., 1999; Benjamin et al., 2010; LeBoeuf et al., 2010). Thus, when made highly relevant, the dominant business culture may prompt a “business norm” and impair interpersonal trust, thus confirming its causal impact on the recent trust crisis.

However, the extent to which business culture impacts interpersonal trust is still unknown. Till date, nearly all the studies that address this question have measured trust with either self-reports or decisions made in trust games. In those measurements, the objects of trust were often abstract or fictitious and lacked crucial facial information. This is important because in general, trustworthiness includes behavioral as well as facial trustworthiness (Xu et al., 2012). The former is determined by characteristics of individual behavior while the latter is determined by human facial features and expressions. People tend to rely on both facial appearance (Zebrowitz, 2005; Willis and Todorov, 2006; Todorov, 2008; Zebrowitz and Montepare, 2008; Cassidy and Gutchess, 2015) and behaviors (McCarthy and Skowronski, 2011) to judge trustworthiness, but faces always take precedence over behavior (Anderson and Barrios, 1961). It has been suggested that judgments about trustworthiness are essential to humans for our safety and survival (Porter et al., 2008). A growing number of studies support the primary role of judgment about facial trustworthiness in forming interpersonal trust. In some trust games, for example, it has been shown that people tend to invest larger amounts of money in an imaginary partner because that partner has higher facial trustworthiness (van't Wout and Sanfey, 2008; Stirrat and Perrett, 2010; Rezlescu et al., 2012).

The judgment of facial trustworthiness is a rapid process that requires an exposure of < 100 milliseconds for decision-making, either in adults or children (Willis and Todorov, 2006; Todorov et al., 2009; Eggleston et al., 2021; Sutherland and Young, 2022). Some research findings suggest that it may be an automatic process. For example, participants in a study were required to make judgments of trustworthiness based on faces with an exposure of only 33 ms, and the outcome was above the level of chance (Todorov et al., 2009). Bonnefon et al. (2013) found that judgments of trustworthiness were not influenced by an increase in cognitive load. FMRI studies have demonstrated that with increased levels of untrustworthiness of faces, the right amygdala responded in a negative linear pattern while the response of the left amygdala was quadratic, although the participants were required to also evaluate the age of the faces or to memorize them (Engell et al., 2007; Todorov et al., 2008). Further evidence suggests that the judgment of facial trustworthiness is a bottom–up process. It was found that four facial characteristics, namely, the brow ridge (down/up), cheekbones (shallow/pronounced), chin (wide/thin), and nose sellion (shallow/deep) are significant predictors of facial trustworthiness (Todorov et al., 2008). Stirrat and Perrett (2010) found that men with wider faces were more likely to exploit others' trust. Rostovtseva et al. (2024) also identified implausible face shapes characterized by relatively narrow jaws and low eyebrow positions.

Therefore, it is of interest to explore whether business culture could impair such rapid, automatic, and bottom–up trustworthiness judgments. If the answer is yes, we can further our understanding of the trust crisis and define specific possible interventions because we cannot easily control or change our judgments of facial trustworthiness. Qi et al. (2018) found that an individual's monthly income can modulate trustworthiness judgments and subsequent trust behavior based on facial appearance, suggesting that judgments of facial trustworthiness may be subject to top–down information. Rostovtseva et al. (2023a) demonstrated that although there were no gender differences in facial trustworthiness toward strangers in complete anonymity, significant gender effects were revealed after viewing the participants' silent video, again suggesting that judgments of facial trustworthiness are influenced by top-down information. Similarly, Rostovtseva et al. (2023b) showed that the presentation of short silent videos influenced participants' judgments of their interaction partners' facial trustworthiness, which also affected their pro-social behavior with their interaction partners. Meanwhile, Chua and Freeman (2020) study demonstrated that trustworthiness-related facial stereotypes can be reshaped through behavioral counterstereotype training. The accompanying study by Chua and Freeman (2022) further showed that there is an implicit learning mechanism for our assessment of facial features, that is, we can learn to dynamically form new facial stereotypes that are automatically activated in processed judgments of facial trustworthiness and thus have an impact. However, to our knowledge till date, there has been no study that has investigated the impact of business culture on facial trustworthiness judgments.

To verify that business culture can have a top-down effect on facial trustworthiness judgments, we designed four experiments. In experiments 1 and 2, we compared college students from business-related and other non-related majors and people working in careers related to business and those from other backgrounds. We adopted classic facial trustworthiness judgment tasks from previous studies in which participants were required to evaluate novel faces on their trustworthiness and categorize those faces as trustworthy or untrustworthy (Todorov et al., 2009). The evaluation scores for trustworthy and untrustworthy faces were compared to determine whether participants were accurate in trustworthiness judgments on faces. To further examine the causal impact of business culture on judgments of facial trustworthiness, we also made use of imaginary scenarios from a first-person perspective to prompt the “business norm”. It is well-known that environmental information affects our judgments and behaviors (Lerner et al., 2015; Powell et al., 2018). For example, witnessing someone's pain and vulnerability triggers emotional and empathic responses that lead to increased altruistic behaviors or other related behaviors (Smith, 2011). Further, vividly portraying the suffering of a single individual is an effective way to encourage donations (Kogut and Ritov, 2005). In addition, the first-person perspective makes imagined events more real and engaging than the third-person perspective (Mcisaac and Eich, 2002; Pronin and Ross, 2006; Sanitioso, 2008). Therefore, in experiments 3 and 4, we asked college students from business-related and other non-related majors, respectively, to imagine the daily work situations of an employee of an investment bank or a non-financial institution using the first-person perspective, followed by the trustworthiness judgment task regarding novel faces. And Experiment 3 we activated the business culture of non-business-related majors, and in Experiment 4 we activated the non-business culture of business-related majors, and the two experiments formed a contrast. Through these four experiments, we aimed to explore whether business culture impairs people's judgment of the trustworthiness of novel human faces.

## 2 Experiment 1

### 2.1 Materials and methods

#### 2.1.1 Participants

We recruited 30 first-year Chinese students majoring in economics or business administration (15 men and 15 women) and 30 third-year Chinese students majoring in humanities and social sciences (15 men and 15 women) to participate in the study. Due to data missing, we cannot report exact demographic information for the participants, but we can confirm that all were between the ages of 20 and 22, with normal or corrected vision.

#### 2.1.2 Materials

Fifty neutral male faces and 50 neutral female faces were selected randomly from the Chinese Facial Affective Picture System (Gong et al., 2011). Before the experiment, the trustworthiness of each face was evaluated by 16 college students (eight men and eight women) on a scale ranging from 1 (extremely untrustworthy) to 7 (extremely trustworthy). Each of those 16 students scored all 100 photographs. We defined the 10 faces with the highest scores as the set of trustworthy faces (five men's faces and five women's faces) and the bottom 10 as the set of untrustworthy faces (five men's faces and five women's faces). The average trustworthiness score of the trustworthy faces (*M* = 4.68, *SD* = 0.20) was significantly higher than that of untrustworthy faces (*M* = 3.36, *SD* = 0.20), *F*_(1, 18)_ = 208.95, *p* < 0.001, $\eta_{p}^{2}$ = 0.921. There was no difference in the attractiveness of trustworthy faces (*M* = 4.16, *SD* = 0.30) and untrustworthy faces (*M* = 4.08, *SD* = 0.23), *F*_(1, 18)_ = 0.37, *p* > 0.05, $\eta_{p}^{2}$ = 0.020.

#### 2.1.3 Procedure

Participants were informed that the study aimed to test their first impression of unknown faces using photographs. After the participants were briefed on the requirements, they were seated comfortably in a dimly lit and sound-attenuating chamber about 80 cm from the computer screen. After a “+” appeared in the center of the screen for 1,000 ms, 10 trustworthy faces and 10 untrustworthy faces appeared randomly for 100 ms, and all 20 faces appeared once. Participants input scores using the computer's numerical keyboard using a scale ranging from 1 to 7. They had up to 10 s to judge the trustworthiness and attractiveness of each face.

### 2.2 Results

**Figure 1:**
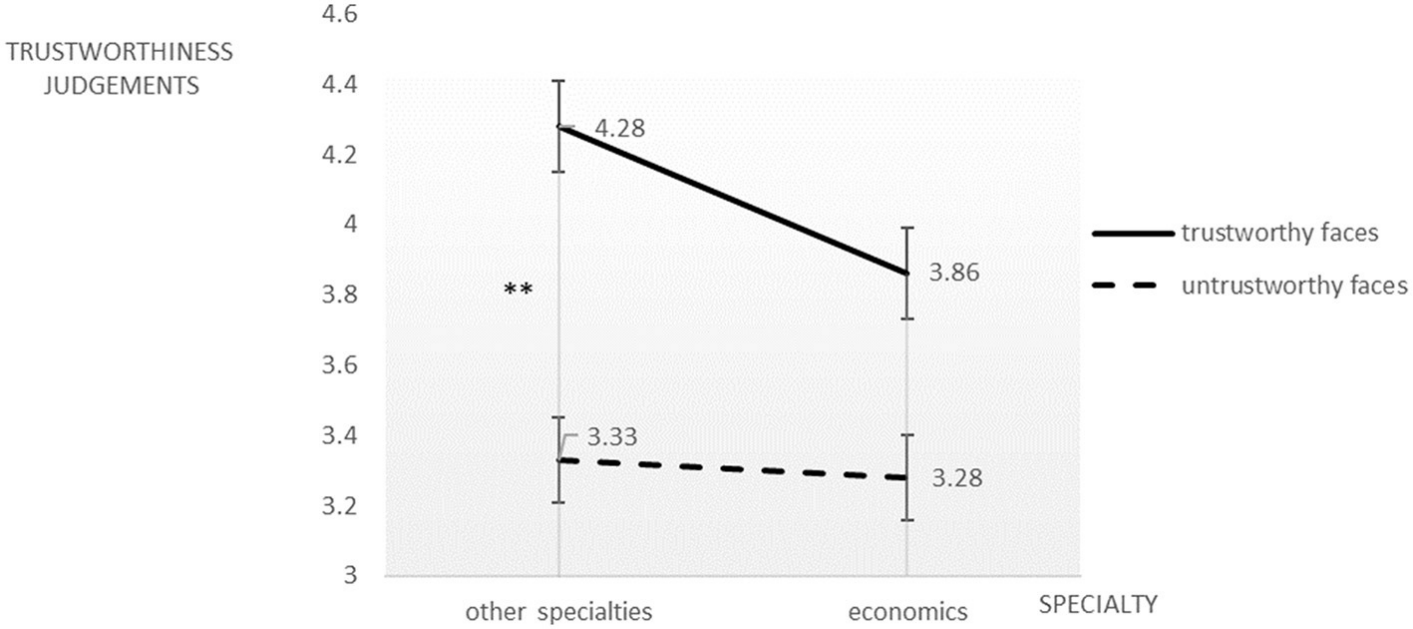
Trustworthiness judgments on novel trustworthy and untrustworthy faces by college students from economics and non-economics majors. **p < 0.01.

We conducted a two (business-related majors vs. non-related majors) × two (trustworthy faces vs. untrustworthy faces) ANOVA with a trustworthiness judgment score as the dependent variable. Participants gave higher trustworthiness scores to trustworthy faces (*M* = 4.07, *SD* = 0.72) than to untrustworthy faces (*M* = 3.31, *SD* = 0.65), *F*_(1, 58)_ = 131.99, *p* < 0.01, $\eta_{p}^{2}$ = 0.695. The effect of the students' majors by itself was insignificant, *F*_(1, 58)_ = 2.17, *p* = 0.146 > 0.05, $\eta_{p}^{2}$ = 0.036. However, the two-way interaction was significant, *F*_(1, 58)_ = 7.61, *p* < 0.01, $\eta_{p}^{2}$ = 0.116. The simple effect analysis revealed that participants from business-related majors gave significantly lower trustworthiness scores to trustworthy faces (*M* = 3.86, *SD* = 0.71) than those from other majors (*M* = 4.28, *SD* = 0.67); but there was no significant difference for untrustworthy faces (Figure 1).

## 3 Experiment 2

### 3.1 Materials and methods

Thirty Chinese bank employees (13 men and 17 women; *M*_*age*_ = 29.3, *SD*_*age*_ = 5.23) and 30 Chinese employees working in non-financial occupations (e.g., social workers, teachers, and others; 14 men and 16 women; *M*_*age*_ = 32.4, *SD*_*age*_ = 11.16) were recruited to participate in the study. They were required to make judgments about facial trustworthiness for the same novel faces that were used in Experiment 1. The only difference was that this time, the faces were printed on a questionnaire, and participants had as much time as they needed to make their evaluations.

## 4 Results

**Figure 2:**
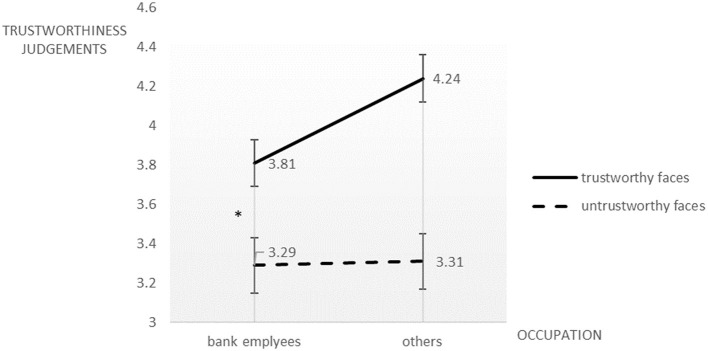
Trustworthiness judgments on novel trustworthy and untrustworthy faces by bank employees and others having non-financial occupations. *p < 0.05.

We performed a two (financial occupations vs. non-financial occupations) × two (trustworthy faces vs. untrustworthy faces) ANOVA with the trustworthiness judgment score as the dependent variable. Similar to Experiment 1, participants gave higher trustworthiness scores to the trustworthy faces than to the untrustworthy faces, *F*_(1, 58)_ = 57.57, *p* < 0.05, $\eta_{p}^{2}$ = 0.498, while the effect of occupation was not significant, *F*_(1, 58)_ = 2.15, *p* > 0.05, $\eta_{p}^{2}$ = 0.036. Again, the two-way interaction was significant, *F*_(1, 59)_ = 4.55, *p* < 0.05, $\eta_{p}^{2}=$ 0.073. A simple effect analysis revealed that employees in the financial sectors awarded significantly lower trustworthiness score to the trustworthy faces (*M* = 3.81, *SD* = 0.61) compared to those in other occupations (*M* = 4.24, *SD* = 0.69) but there was no significant difference between the two groups for untrustworthy faces (Figure 2).

## 5 Experiment 3

### 5.1 Materials and methods

We recruited 61 Chinese college students from non-business-related majors (37 men and 24 women; *M*_*age*_ = 18.4, *SD*_*age*_ = 0.74) to participate in the third experiment. The students were randomly assigned to one of two scenarios: in the first scenario, they were told to imagine themselves being an investment bank employee; in the other scenario, they were told to imagine themselves in the role of clerical staff. They were required to read and transcribe a short message (142 words in both scenarios) describing either a bank employee or a clerical staff's daily life in the workplace. In this way, we aimed to simulate the exposure to the business culture vs. non-business culture. After this task, participants were required to make judgments about facial trustworthiness using the same procedures and materials as in Experiment 1.

### 5.2 Results

**Figure 3:**
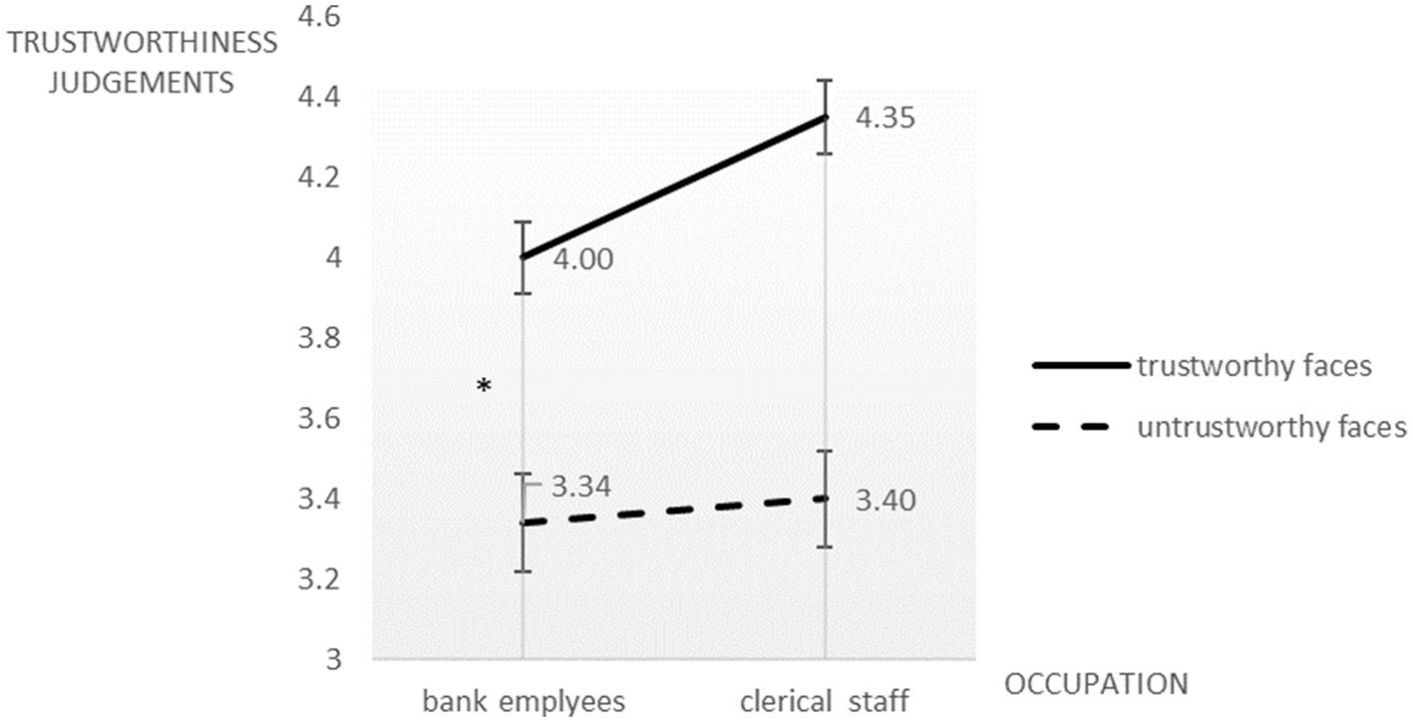
Trustworthiness judgments on novel trustworthy and untrustworthy faces by college students from non-economic majors who imagined themselves being an investment bank employee or a clerical staff. ^*^p < 0.05.

We performed a two (bank employee vs. clerical staff) × two (trustworthy faces vs. untrustworthy faces) ANOVA with the trustworthiness judgment score as the dependent variable. Participants still gave higher trustworthiness score to the trustworthy faces than to the untrustworthy faces, *F*_(1, 59)_ = 132.37, *p* < 0.05, $\eta_{p}^{2}$ = 0.692 while the main effect of occupation imagination was not significant, *F*_(1, 59)_ = 2.75, *p* > 0.05, $\eta_{p}^{2}$ = 0.045. Again, the two-way interaction was significant, *F*_(1, 59)_ = 4.33, *p* < 0.05, $\eta_{p}^{2}$ = 0.068. A simple effect analysis revealed that participants who imagined themselves as bank employees awarded significantly lower trustworthiness scores to the trustworthy faces (*M* = 4.00, *SD* = 0.52), than those imagining themselves as clerical staff (*M* =4.35, *SD* = 0.41); but there was no significant difference between the two groups for the untrustworthy faces (Figure 3).

## 6 Experiment 4

### 6.1 Materials and methods

We recruited 63 Chinese college students majoring in business-related majors (19 men and 44 women; *M*_*age*_ = 20.52, *SD*_*age*_ = 2.95) to participate in the fourth experiment. As in Experiment 3, they were randomly assigned to one of two scenarios. They were also required to read and transcribe the same short messages. After that, participants repeated the same trustworthiness judgment procedure as in Experiment 1.

### 6.2 Results

**Figure 4:**
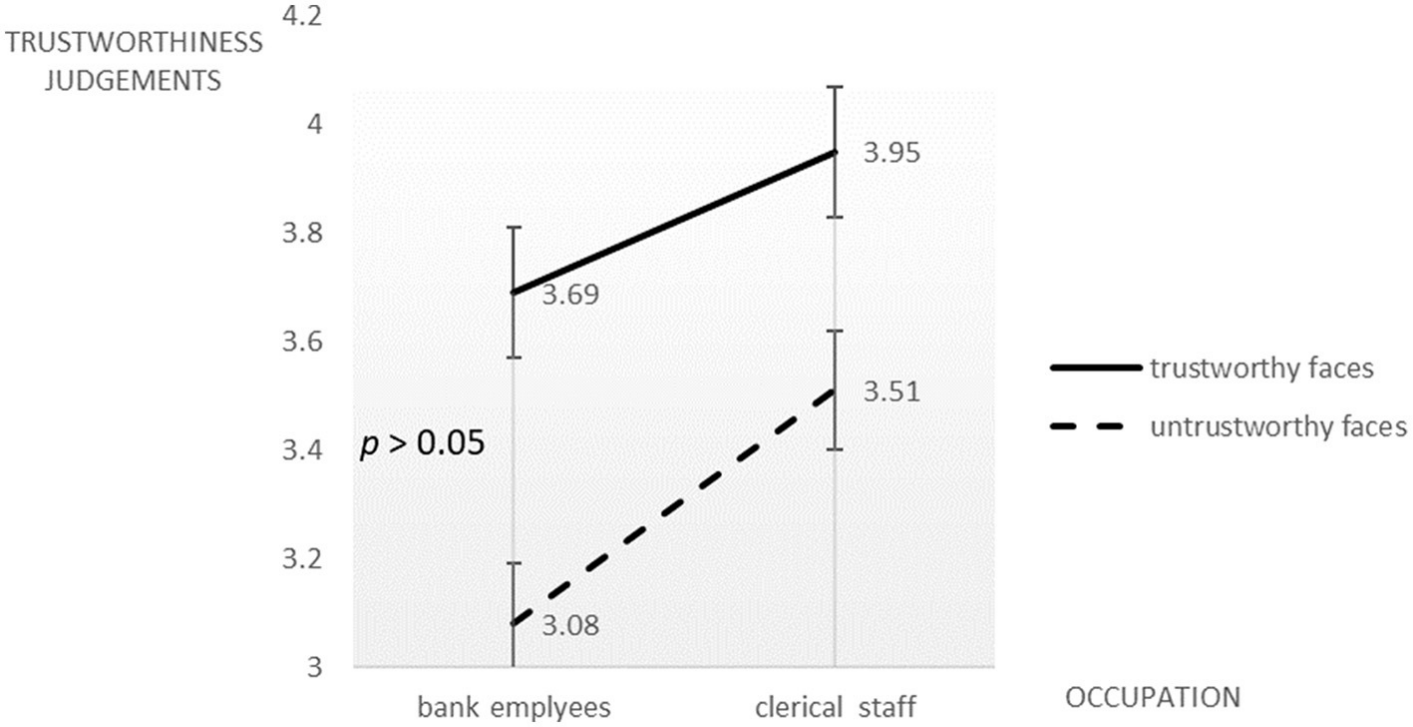
Trustworthiness judgments on novel trustworthy and untrustworthy faces by college students majoring in economics who imagined themselves being an investment bank employee or a clerical staff.

We performed a two (bank employee vs. clerical staff) × two (trustworthy faces vs. untrustworthy faces) ANOVA with the trustworthiness judgment score as the dependent variable. Again, participants gave higher trustworthiness score to trustworthy faces than to untrustworthy faces, *F*_(1, 61)_ = 49.75, *p* < 0.001, $\eta_{p}^{2}$ = 0.45. However, in contrast to previous studies, the primary effect of occupation was significant, *F*_(1, 61)_ = 5.81, *p* < 0.05, $\eta_{p}^{2}$ = 0.09. Those who imagined themselves as clerical staff gave significantly higher trustworthiness scores to both the trustworthy faces (*M*_*bankemployee*_ = 3.69, *SD*_*bankemployee*_ = 0.71; *M*_*clericalstaff*_ = 3.95, *SD*_*clericalstaff*_ = 0.60) and the untrustworthy faces (*M*_*bankemployee*_ = 3.08, *SD*_*bankemployee*_ = 0.67; *M*_*clericalstaff*_ = 3.51, *SD*_*clericalstaff*_= 0.54). Also in contrast to the previous experiments, the two-way interaction was insignificant (*p* > 0.05, Figure 4).

## 7 Discussion

Through four experiments, we confirmed the impact of the prevailing business culture on the trustworthiness judgments of novel faces. In Experiment 1, we compared college students from business-related and non-related majors while in Experiment 2, we compared bank employees with those from non-financial occupations. Both experiments confirmed that those who were more exposed to business culture tended to assign lower trustworthiness scores to trustworthy faces. In Experiment 3, we used a first-person perspective priming for college students from non-business-related majors. By manipulating participants' imagined exposure to business culture, we found the same pattern as in the previous experiments. In Experiment 4, we used the same priming procedure as in Experiment 3 with college students from business-related majors. This time, the imagined exposure to business culture impaired trustworthiness judgments for trustworthy faces and extended even to untrustworthy faces. In all, these findings confirm the hypothesis that business culture has a detrimental impact on judgments of facial trustworthiness, a process formerly believed to be rapid, automatic, and bottom–up.

To the best of our knowledge, this study is the first attempt to show that business culture has such a significant impact on interpersonal trust. In previous studies, trust was measured with self-reported questionnaires or trust games, both believed to incorporate deliberate thinking and often occurring over an extended period. This may allow some cognitive biases such as the subjects' salient business identities to emerge and affect their responses. However, judgments of facial trustworthiness are formed in no longer than 100 ms (Willis and Todorov, 2006; Todorov et al., 2009). It is thus a reasonable guess that the business norm induced by business culture may also be rapid and automatic. Therefore, we may not be able to prevent it from occurring, making interventions difficult. Future research will be needed to address the impact of business culture and its remedies.

The present study does not explore possible mechanisms underlying the impairment of participants' trustworthiness judgments due to exposure to the business culture, either in practice or by imagined scenarios. FMRI studies have revealed that the bilateral amygdala may be responsible for the detection of trustworthiness in faces, although the two sides exhibit differing patterns (Todorov et al., 2008). One imaging study found that older adults differed from younger adults in perceiving untrustworthy faces to be significantly more trustworthy. However, bilateral amygdala responses showed no significant differences between the two age groups. Instead, older adults showed muted activation of the anterior insula when viewing untrustworthy faces (Castle et al., 2012). The authors linked the muted anterior insula activation to a diminished “gut feeling” regarding facial untrust cues rather than the emotional threat detected by the amygdala. It is possible that in contrast to older adults, participants who are more exposed to business culture may have an amplified interoceptive awareness of facial untrust cues.

How did this amplified awareness of facial untrust cues occur? Previous studies have proposed several possible mediating mechanisms. First, the line of occupational identity research proposes that business culture promotes unethical behaviors and leads employees to have less trust in others because of its focus on materialistic values (Cohn et al., 2014). However, in our experiments, participants were presented with novel faces they had never seen, leaving materialistic cues (such as attire) to a minimum. It is difficult to believe that our participants were affected by materialistic values in showing distrust toward novel faces. Second, Zhong (2011) observed that participants engaging in a deliberative task showed reduced altruistic motivation on subsequent moral judgment tasks. Indeed, Belmi and Pfeffer (2015) suggest that people feel less obligated to reciprocate in an organizational context. Kouchaki et al. (2013) also found that money cues triggered a business decision frame, which led to a greater likelihood of unethical intentions and behavior afterwards. However, the rapid formation of judgments regarding facial trustworthiness in the present study seems to leave no room to engage in any kind of deliberative or calculative processes. Lastly, we propose that an implicit *Homo economicus belief* may influence the formation of novel facial stereotypes, leading to more positive trustworthiness judgments of new faces. The *Homo economicus belief* represents an assumption in mainstream economics about human nature that people always aim to maximize their interests (Xin and Liu, 2013; Yamagishi et al., 2014; Chen and Liu, 2017). If our belief system operates this way, we would become more cautious about others and, thus, may overweigh untrust cues in evaluating the trustworthiness of novel faces. This corroborates Chua and Freeman (2020) study that novel facial stereotypes are automatically activated early in the processing of facial trustworthiness judgments, thus biasing participants' trust behavior.

In Experiment 4, we found that, after being primed with business culture, college students from business-related majors exhibited a similar pattern of impaired judgment toward trustworthy faces, consistent with previous findings. However, the same impaired pattern was also found for untrustworthy faces, suggesting that the detrimental impact of business culture may be cumulative. It also raises the question of whether by inducing a counterbalancing culture or identity, we can mitigate the detrimental impact of business culture. Participants in a study were asked to read a paragraph about a family business's net worth to induce a calculative mindset. They found that compared to the non-calculative tasks, this led people to be consistently more selfish in the Dictator Game (Wang et al., 2014). However, a subtle intervention such as choosing among four family photographs to be the target of the family business diminished the effects of the calculative task (Wang et al., 2014). Future research is needed to examine whether similar interventions can improve judgments of facial trustworthiness.

In conclusion, the present study found that following real or imagined exposure to business culture, participants exhibited impaired judgment regarding the trustworthiness of novel faces that had been identified previously as trustworthy.

## Data availability statement

The original contributions presented in the study are included in the article/supplementary material, further inquiries can be directed to the corresponding author.

## Ethics statement

The studies involving humans were approved by Institutional Review Board of Central University of Finance and Economics. The studies were conducted in accordance with the local legislation and institutional requirements. The participants provided their written informed consent to participate in this study.

## Author contributions

HZ: Conceptualization, Data curation, Formal analysis, Funding acquisition, Methodology, Supervision, Validation, Writing – review & editing. YL: Data curation, Writing – review & editing. WL: Writing – original draft. MN: Data curation, Formal analysis, Visualization, Writing – review & editing. ZX: Conceptualization, Writing – review & editing.
